# Early-Life Exposure to the Chinese Famine Is Associated with Higher Methylation Level in the *INSR* Gene in Later Adulthood

**DOI:** 10.1038/s41598-019-38596-6

**Published:** 2019-03-04

**Authors:** Zhenghe Wang, Jieyun Song, Yanhui Li, Bin Dong, Zhiyong Zou, Jun Ma

**Affiliations:** School of Public Health & Institute of Child and Adolescent Health, Peking University, Beijing, China

## Abstract

We examined the association between the China famine exposure in early life and DNA methylation of *INSR* (hg18, chr19:7110130-7110574) and *CPT1A* (hg18, chr11: 68286513-68286952) related to growth and metabolism in 235 subjects selected from two provinces in China. The subjects were categorized into prenatal famine-exposed group and non-exposed group based on their birthdates. DNA methylation at the *INSR* gene locus was assayed from peripheral white blood cells using the Sequenom’s MassARRAY system. Two dependent samples *t*-test was used to compare the difference between the exposed group and non-exposed group. DNA methylation level of *INSR* was higher among individuals who exposed to the China famine in the fetus than that of non-exposed group (*d* = 3.3%, *P* = 0.006). A significant interaction between famine exposure and province was observed for *INSR* (*P*_interaction_ < 0.001). DNA methylation level of *INSR* was positively associated with triglyceride (*β* = 0.011, *P* = 0.021), and negatively associated with high-density lipoprotein cholesterol (*β* = −0.039, *P* = 0.021). Moreover, exposed group had higher meat consumption than non-exposed group in severe exposure area. Prenatal exposure to the China famine plus later life eating habits might regulate epigenome.

## Introduction

Intrauterine growth retardation is associated with increased risk of chronic non-communicable diseases (NCDs)^1,2^. Studies have observed that participants who born with low birth weight always have higher risk of type 2 diabetes mellitus^3^, hypertension^4^, and obesity^5^ in later life. These associations were supported by the theory of the Developmental Origins of Health and Disease (DOHaD) that fetus exposure to limited nutrition condition could induce permanent alterations in the offspring’s structure and function to adapt poor intrauterine environment^6^. These adaptation changes could be helpful for surviving but may be harmful for later health condition. The direct evidence in human beings that severe malnutrition exposure in fetal stage increased the risk of chronic NCDs came from the Dutch famine study. A study based on 41,096 medical examination records for military service in Netherlands found that prenatal famine exposure did not increase the mortality from cardiovascular diseases^7^. However, another study used the data from the fifth cycle of the Longitudinal Aging Study Amsterdam found that people whose mother exposed to the famine in their fetal stage was associated with higher risks of cardiovascular diseases^8^. In addition, as the largest famine in human history, the China famine also provided important evidences for the hypothesis of DOHaD. Although the China famine lasted for about three years and lead to hundreds of thousands of premature deaths, little attention was paid until 1980 when several researchers analyzed the China Population Census Data and found that more than 30 million people were “missing” from the famine-year (1959–1961) birth cohort^9,10^. Since then, more and more studies shed light on the long-term effects of the famine exposure in survivors’ health. Majority of these studies used the national large-scale epidemiological surveys, such as the China National Nutrition and Health Survey, the China Health and Retirement Longitudinal Survey observed that the famine exposure both in the fetus and early childhood were associated with the elevated risks of NCDs, including hypertension^11,12^, dyslipidemia^13^, type 2 diabetes mellitus^12^, and metabolic syndrome^14^.

The altered DNA methylation of genes related to growth and metabolic may play an important role in leading to such effect. Animal models have shown that dietary protein restriction during prenatal period could decrease the DNA methylation level in promoters regions of the glucocorticoid receptor (*GR*) and peroxisomal proliferator-activated receptor (*PPAR*) genes in rat offspring, and leads to persistent phenotypes changes^15^, which could be transmitted even to the next generation^16–18^. These patterns were also detected in humans according to the studies of the Dutch famine^19,20^.

*INSR* (insulin receptor) influences growth and insulin signaling, and *CPT1A* (carnitine palmitoyltransferase 1A) influences fatty acid oxidation. Recently, a genome-scale analysis identified prenatal malnutrition-associated different methylation regions (P-DMRs) in the Dutch study and observed that DNA methylation level in the intragenic enhancer regions of *INSR* and *CPT1A* were associated with both prenatal famine exposure and serum low-density lipoprotein cholesterol^19^. However, the Bangladesh famine study did not observe the statistically significant difference in DNA methylation level of *INSR* and *CPT1A* between the prenatal famine-exposed group and non-exposed group^21^. China famine are more severe than the Dutch famine and Bangladesh famine. China famine lasted for three years, affected 600 million population and lead to about 30 million premature deaths^9,10^. However, Dutch famine lasted for six months^19^ and Bangladesh famine lasted for 12 months^21^. Thus, China famine could be more valuable to examine the association of early-life the famine exposure with epigenetic features in adulthood. To our knowledge, no study has been designed to explore the relationship between prenatal exposure to China famine and alteration of DNA methylation in later years.

To this end, two common candidate genes related to growth and metabolic, *INSR* and *CPT1A*, were selected in the present study and the associations between China famine exposure in fetus and DNA methylation in adulthood were explored.

## Methods

### Subjects and Data Collection

Subjects in this study were selected from two provinces (Anhui and Jiangxi) in China. According to previous studies of China famine^11,12^, excess mortality was used to reflect the severity of famine exposure because the direct indicators, such as daily calories, birth weight, and birth length, for assessing the severity of famine exposure are unavailable. Excess mortality was calculated as the percentage change of the average mortality rate from pre-famine period (1956–1958) to the average mortality rate during famine (1959–1961). The excess mortality reaches up to 474.9% in Anhui province, which is the most severity province of the China famine exposure. However, excess mortality in Jiangxi province is 36.8%, which is obviously lower than average level in China mainland (122.8%)^22^.

**Figure 1:**
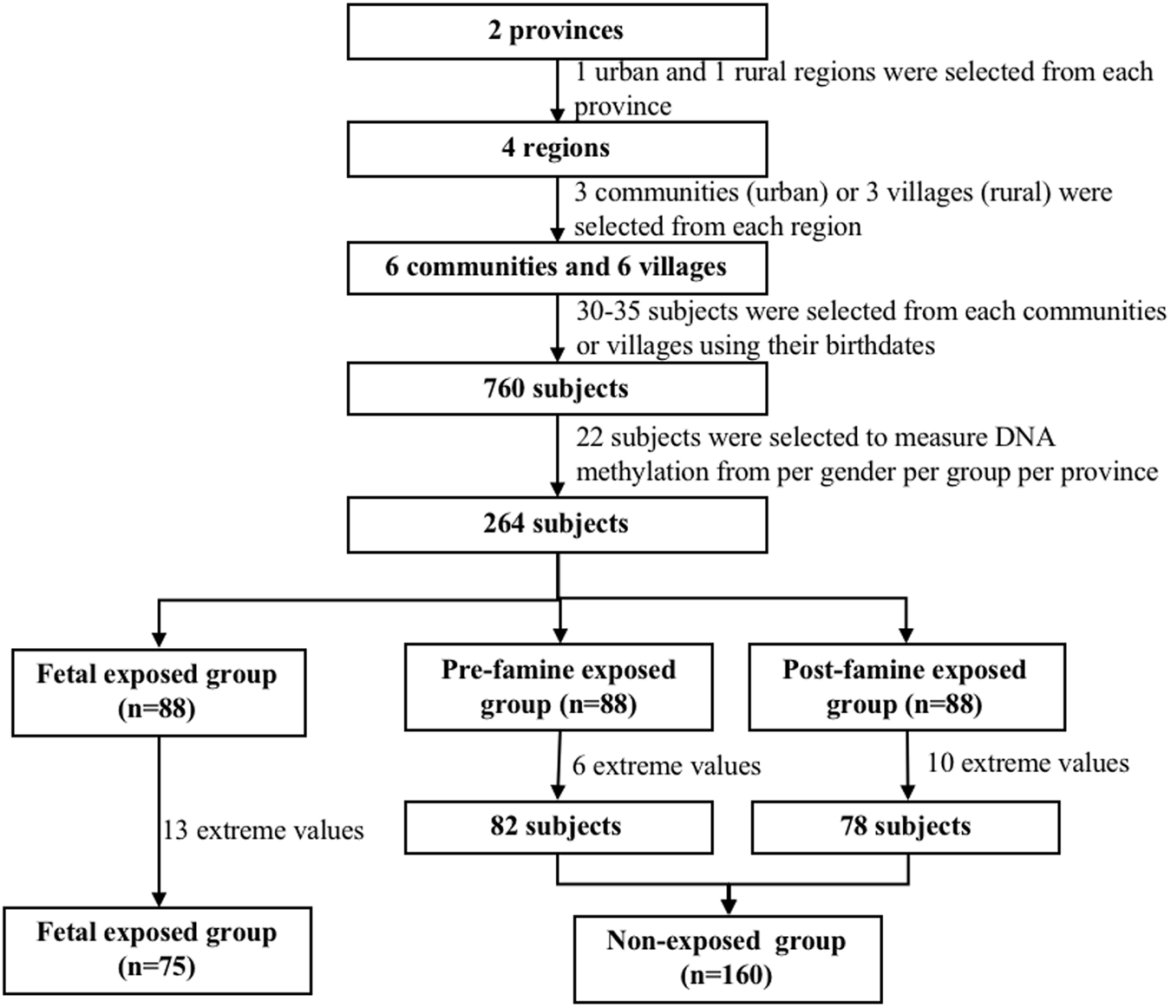
Flow chart on the subjects selecting methods at each step.

The method of multi-stage random sampling was used to collect subjects. The first step was to select randomly 1 urban and 1 rural area from Anhui and Jiangxi provinces, respectively. The second step randomly selected 3 communities and 3 natural villages from each area. The third step randomly selected 30 to 35 subjects in each community or natural village from the resident registration system. Finally, 760 subjects were selected to complete anthropometric measurements, questionnaires and blood sample collection in local community hospital (Fig. 1).

Methylation data was collected from 264 subjects selected randomly from three groups (22 subjects per gender per group per province). After excluding 29 subjects with extreme values of the DNA methylation (>3 standard deviation), 235 subjects were finally enrolled into the analysis (Fig. 1).

As described in previous studies^12^, birthdates of subjects were used to define the famine exposed group and non-exposed group. Subjects who born between October 1^st^, 1959 and September 30^th^, 1961 were categorized as the famine exposed group, subjects who born between January 1^st^ 1958 and December 31^st^, 1958 were categorized as the pre-famine exposed group, and subjects who born between October 1^st^, 1962 and September 30^th^, 1964 were categorized as the post-famine exposed group. We combined the pre-famine exposed group and post-famine exposed group as the non-exposed group. Due to the China famine lasted from Autumn of 1958 to Autumn of 1962, especially during 1959–1961, subjects who born between October 1st 1961 and September 30th 1962 were excluded in the current study because they might also exposure to famine during second or third trimester of pregnancy.

### Anthropometric measurements and questionnaire

Anthropometric measurements, including height, weight, and waist circumference, were measured using a standard procedure. Blood pressure was measured using a column Mercury Sphygmomanometer (model XJ11D, China) in right arm. Fasting plasma glucose (FPG) and lipid profiles were measured by a qualified biomedical analyses company with the method of enzymatic colorimetric test.

The International Physical Activity Questionnaire Short Form (IPAQ-SF) was used to collect information of physical activity (PA), and participants were categorized as vigorous PA, moderate PA, or light PA group. We combined the moderate PA and light PA group as the non-vigorous PA group. Food frequency questionnaire was used to collect dietary data, and the consumption frequencies of meat, vegetables, fruit, and milk were categorized into two groups (every day and non-daily). Self-reported smoking status was classified into two groups, subjects who smoked <400 cigarettes in lifetime, and who smoked ≥400 cigarettes in lifetime. Alcohol consumption was also reported by participants and was classified into one of two categories (subjects who drank less than once a month, or who drank not less than once a month in the past year).

This project has been approved by the Ethics Committee of Peking University Health Science Center (IRB00001052-15057), and all the participants signed the written informed consent form. All methods were performed in accordance with the relevant guidelines and regulations.

### DNA Methylation Measurements

The procoagulant peripheral whole blood samples were collected by the trained registered nurse. The method of salting out was used to extract genome DNA from blood clots. The EZ 96- methylation kit (Zymo Research) was used to bisulfite converted genomic DNA lasting for 5.5 hours in 37 °C. The Sequenom’s MassARRAY system (Sequenom, San Diego, CA) was used to quantitatively assessed DNA methylation using the manufacturers’ protocol on the 384-well plate, which has been widely used to perform DNA methylation assessments^23,24^. The positions of hg18, chr19:7110130-7110574 in intragenic enhancer region of *INSR* and hg18, chr11:68286513-68286952 in promoter region of *CPT1A* were amplified and measured DNA methylation. The primers of *INSR* and *CPT1A* were designed using the Epidesigner online application (http://epidesigner.com/start3.html). The details of primers were presented in Table S1.

### Statistical analyses

The SPSS 20.0 (IBM, Chicago, IL, USA) were used to performed statistical analysis. The continuous variables were presented as mean (standard deviation), categorical variables were presented as numbers (proportion). The quantile-quantile plot was used to test normality of continuous variables. Independent *t*-test was used to compare the differences of continuous variables between exposed group and non-exposed group, and the Cohen’s *d* equation was used to calculate the effect size (*z*). The method of Bonferroni was used to correct the multiple comparisons, and the Chi-square test was used to analyze the difference for categorical variables.

To examine whether the DNA methylation differences between exposed group and non-exposed group was region-specific, we performed stratified analysis by region. Moreover, an interaction analysis was also examined by adding a multiplicative factor in the general linear model.

Linear regression was used to analyze the associations between DNA methylation and main phenotypes.

A power analysis was performed to assessed the statistical power for examining the difference of DNA methylation between the famine exposed group and non-exposed group. The mean methylation level of *INSR* was 0.434 and 0.467 in the non-exposed group and prenatal exposed group, respectively, and the standard deviation was 0.092 and 0.070, respectively. We set the significance level as 0.05 with a two-tailed test. Because 75 famine-exposed subjects and 160 non-exposed subjects were enrolled into the analysis. A power of 0.855 was evaluated for examining the DNA methylation difference between the fetal-exposed group and non-exposed group.

## Results

### The basic characteristics of the study population

A total of 235 participants were enrolled into the present study (Table 1). The mean age of the exposed group and non-exposed group was 55.1 and 54.6 years, respectively. Subjects who exposed to the China famine in fetal stage had higher waist circumference (*d* = 2.89, *P* = 0.029) and current drinking rate (47.7% vs. 34.2%, *P* = 0.035) than those who did not exposed to the famine. However, we did not observe any significant differences for other factors between exposed group and non-exposed group (*P* > 0.05). When further stratified by province. We found that only severe famine exposure had higher levels of the WC and FPG and higher frequency of alcohol use than non-exposed group (*P* < 0.05). However, consistent results were not observed in less severe areas. In addition, exposed group had higher frequency of meat intake than that in the non-exposed group in severe famine exposure areas, but the difference was not statistically significant (*P* > 0.05) (Table 2).

**Table 1:** The basic characteristics of study population.

Variables	Exposed group (n = 75)	Non-exposed group (n = 160)	*t*/*χ*^2^	*P*
Age (mean ± SD), years	55.1 (0.8)	54.6 (2.6)	1.45	0.149
Height (mean ± SD), cm	160.79 (7.77)	160.86 (8.29)	0.06	0.949
Weight (mean ± SD), kg	63.27 (10.1)	62.77 (10.83)	0.36	0.720
WC (mean ± SD), cm	85.86 (10.57)	82.97 (9.78)	2.20	0.029
BMI (mean ± SD), kg/m^2^	24.43 (3.2)	24.27 (3.85)	0.34	0.737
FPG (mean ± SD), mmol/L	6.12 (2.17)	5.68 (1.41)	1.69	0.094
TG (mean ± SD), mmol/L	1.78 (1.05)	1.77 (1.13)	0.08	0.932
HDL-C (mean ± SD), mmol/L	3.05 (0.89)	2.87 (0.77)	0.08	0.934
LDL-C (mean ± SD), mmol/L	1.27 (0.34)	1.27 (0.33)	1.64	0.102
DBP (mean ± SD), mmHg	83.1 (11.5)	82.57 (10.55)	0.37	0.710
SBP (mean ± SD), mmHg	127.92 (18.04)	128.82 (17.24)	0.39	0.694
PA level n (%)			1.281	0.258
Non-vigorous	69 (80.2)	136 (73.9)		
Vigorous	17 (19.8)	48 (26.1)		
Meat n (%)			0.227	0.634
Non-daily	54 (62.8)	121 (65.8)		
Every day	32 (37.2)	63 (34.2)		
Vegetable n (%)			1.933	0.164
Non-daily	8 (9.3)	9 (4.9)		
Every day	78 (90.7)	175 (95.1)		
Fruit n (%)			0.045	0.831
Non-daily	54 (62.8)	118 (64.1)		
Every day	32 (37.2)	66 (35.9)		
Milk n (%)			0.000	0.983
Non-daily	73 (84.9)	156 (84.8)		
Every day	13 (15.1)	28 (15.2)		
Smoking n (%)			1.047	0.306
<400 cigarettes	52 (60.5)	123 (66.8)		
≥400 cigarettes	34 (39.5)	61 (33.2)		
Drinking n (%)			4.467	0.035
<Once per month	45 (52.3)	121 (65.8)		
≥Once per month	41 (47.7)	63 (34.2)		

Abbreviations: WC, waist circumference; BMI, body mass index; FPG, fasting plasma glucose; TG, triglyceride; HDL-C, high-density lipoprotein cholesterol; LDL-C, low-density lipoprotein cholesterol; DBP, diastolic blood pressure; SBP, systolic blood pressure; PA, physical activity.

Independent-samples *t*-test was used to compare difference of continuous variables between exposed group and non-exposed group, and Chi-square test was used to compare the difference of categorized variables between exposed group and no-exposed group.

**Table 2:** The comparison of basic characteristics of study population stratified by areas.

Variables	Anhui		Jiangxi	
	Non-exposed group	Exposed group	Non-exposed group	Exposed group
Age (mean ± SD), years	54.9 (2.7)	55.3 (0.6)	54.5 (2.6)	54.8 (0.9)
Height (mean ± SD), cm	160.93 (7.66)	160.83 (7.71)	160.13 (8.76)	160.75 (8.15)
Weight (mean ± SD), kg	65.21 (10.52)	66.15 (9.65)	60.21 (10.86)	60.11 (9.74)
WC (mean ± SD), cm	84.51 (9.91)	87.56 (8.44)*	81.83 (9.51)	83.98 (12.34)
BMI (mean ± SD), kg/m^2^	25.11 (3.19)	25.53 (2.95)	23.56 (4.35)	23.22 (3.06)
FPG (mean ± SD), mmol/L	5.96 (1.26)	6.70 (2.28)*	5.42 (1.53)	5.47 (1.87)
TG (mean ± SD), mmol/L	1.83 (1.19)	2.04 (1.23)	1.73 (1.08)	1.49 (0.73)
HDL-C (mean ± SD), mmol/L	1.37 (0.34)	1.33 (0.37)	1.17 (0.28)	1.20 (0.31)
LDL-C (mean ± SD), mmol/L	3.12 (0.80)	3.35 (0.93)	2.62 (0.66)	2.71 (0.70)
DBP(mean ± SD), mmHg	86.93 (8.49)	87.36 (9.70)	77.61 (10.29)	78.31 (11.60)
SBP (mean ± SD), mmHg	136.88 (16.13)	136.78 (14.55)	120.53 (14.42)	117.95 (16.42)
PA level n (%)				
Vigorous	74 (82.2)	40 (88.9)	59 (67.0)	29 (70.7)
Non-vigorous	16 (17.8)	5 (11.1)	29 (33.0)	12 (29.3)
Meat n (%)				
Non-daily	58 (64.4)	24 (53.3)	59 (67.0)	30 (73.2)
Every day	32 (35.6)	21 (46.7)	29 (33.0)	11 (26.8)
Vegetable n (%)				
Non-daily	2 (2.2)	3 (6.7)	7 (8.0)	5 (12.2)
Every day	88 (97.8)	42 (93.3)	81 (92.0)	36 (87.8)
Fruit n (%)				
Non-daily	50 (55.6)	22 (48.9)	62 (70.5)	32 (78.0)
Every day	40 (44.4)	23 (51.1)	26 (29.5)	9 (22.0)
Milk n (%)				
Non-daily	73 (81.1)	38 (84.4)	78 (88.6)	35 (85.4)
Every day	17 (18.9)	7 (15.6)	10 (11.4)	6 (14.6)
Smoking n (%)				
<400 cigarettes	58 (64.4)	27 (60.0)	65 (73.9)	25 (61.0)
≥400 cigarettes	32 (35.6)	18 (40.0)	23 (26.1)	16 (39.0)
Drinking n (%)				
<Once per month	56 (62.2)	19 (42.2)	63 (71.6)	26 (63.4)
≥Once per month	34 (37.8)	26 (57.8)*	25 (28.4)	15 (36.6)

Abbreviations: WC, waist circumference; BMI, body mass index; FPG, fasting plasma glucose; TG, triglyceride; HDL-C, high-density lipoprotein cholesterol; LDL-C, low-density lipoprotein cholesterol; DBP, diastolic blood pressure; SBP, systolic blood pressure; PA, physical activity.

Independent-samples *t*-test was used to compare difference of continuous variables between exposed group and non-exposed group, and Chi-square test was used to compare the difference of categorized variables between exposed group and no-exposed group. *Represents *P* < 0.05.

### Association of prenatal famine exposure with DNA methylation

**Figure 2:**
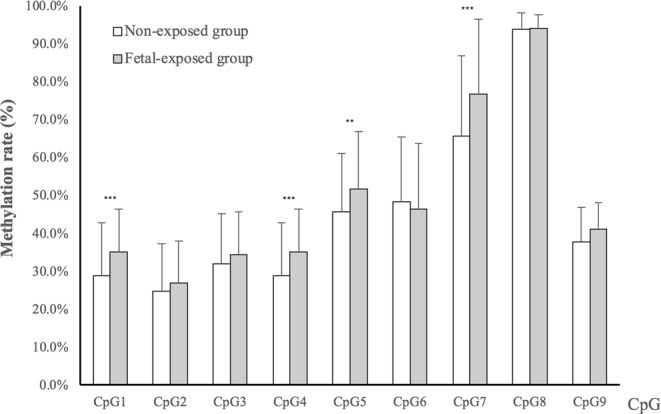
DNA methylation difference between fetal-exposed group and non-exposed group at each CpG unit of the loci *INSR*. The amplicon comprised 9 CpG sites at the *INSR* differential methylation region in Human Genome 18 assembly- chr19: 7,110,130-chr 19: 7,110,574 (NCBI 36/hg18). The location of each CpG site was listed below: CpG1:7,110,208; CpG2: 7,110,217; CpG3: 7,110,260; CpG4: 7,110,345; CpG5: 7,110,361; CpG6: 7,110,389; CpG7: 7,110,423; CpG8: 7,110,454; CpG9: 7,110,540. Independent-samples *t*-test to compare the DNA methylation difference between exposed group and non-exposed group. **Presents *P* < 0.01, ***presents *P* < 0.001.

**Figure 3:**
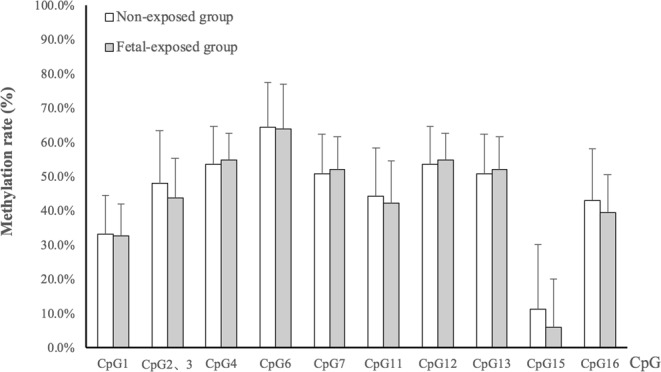
DNA methylation difference between fetal-exposed group and non-exposed group at each CpG unit of the loci *CPT1A*. The amplicon comprised 9 CpG sites at the *INSR* differential methylation region in Human Genome 18 assembly- chr11: 68,286,513 - chr11: 68,286,952 (NCBI 36/hg18). The location of each CpG site was listed below: CpG1: 68,286,641; CpG2&3: 68,286,671&68,286,680; CpG4: 68,286,696; CpG6: 68,286,706; CpG7: 68,286,729; CpG11: 68,286,762; CpG12: 68,286,778; CpG13: 68,286,833; CpG15: 68,286,894; CpG16: 68,286,925. Independent-samples *t*-test to compare the DNA methylation difference between exposed group and non-exposed group.

DNA methylation differences of *INSR* and *CPT1A* between exposed group and non-exposed group are presented in Table 3, Figs 1 and 2. The mean methylation rates of *INSR* and *CPT1A* were 44.1 ±7.4% and 44.8 ± 7.7%, respectively. Compared with the non-exposed group, fetal-exposed group had higher DNA methylation of *INSR* (*d* = 3.3%, *P* = 0.003), even after the Bonferroni correction (*P* = 0.006). Similar associations were also observed at units of CpG 1, CpG 4, CpG 5, and CpG7 (*P* < 0.01) (Fig. 2). However, we did not observe significant difference in mean DNA methylation level and each CpG unit of *CPT1A* between exposed and non-exposed individuals (*d* = −1.4%, *P* = 0.605) (Fig. 3).

**Table 3:** DNA methylation (%) and fetal stage exposure to famine

Genes locus	Exposed group (n = 75)	Non-exposed group (n = 160)	*d* ^a^	Effect size	*P*-value^b^	*P*-value^c^
*INSR*	46.7 ± 7.0	43.4 ± 9.2	3.3	0.20	0.003	0.006
*CPT1A*	44.1 ± 7.8	45.5 ± 8.8	−1.4	−0.08	0.605	NS^d^

Abbreviations: *INSR*, insulin receptor; *CPT1A*, carnitine palmitoyltransferase 1A.

^a^Average absolute difference in DNA methylation between exposed group and non-exposed group.

^b^Linear regression models were used to compare the difference between exposed group and non-exposed group.

^c^Bonferroni-corrected *P*-value.

^d^No significance.

### Province-specific associations

The association of DNA methylation with famine exposure stratified by province is presented in Table 4. In Anhui province, prenatal exposure to the famine is associated with the elevated the DNA methylation of *INSR* (*d* = 2.6%, *P* = 0.018), but not for *CPT1A* (*d* = −2.2%, *P* = 0.1.41). However, we did not observe any statistically significant association between DNA methylation and famine exposure in Jiangxi province (*P* > 0.05). In addition, the interaction between prenatal famine exposure with region was detected for *INSR* (*P*_*Interaction*_ < 0.001), but not for *CPT1A* (*P*_*Interaction*_ = 0.926).

**Table 4:** DNA methylation and fetal stage exposure to famine stratified by region.

Genes locus	Exposed group	Non-exposed group	*d* ^a^	Effect size	*P*-value^b^	*P-*value^c^
*INSR*						
Anhui	46.2 ± 4.7	43.5 ± 7.4	2.6	0.21	0.018	<0.001
Jiangxi	47.3 ± 9.1	43.3 ± 10.9	4.0	0.20	0.062	
*CPT1A*						
Anhui	44.6 ± 7.4	46.8 ± 7.6	−2.2	−0.15	0.141	0.926
Jiangxi	41.2 ± 10.0	43.9 ± 10.0	−2.7	−0.13	0.548	

Abbreviations: *INSR*, insulin receptor; *CPT1A*, carnitine palmitoyltransferase 1A.

^a^Average absolute difference in DNA methylation between exposed group and non-exposed group.

^b^Independent-samples *t*-test to compare the difference between exposed group and non-exposed group.

^c^Interaction analysis between region and famine exposure.

### Association between DNA methylation and phenotypes

Table 5 presents the association between DNA methylation level and common phenotypes. Increased DNA methylation level in intragenic enhancer region of *INSR* was associated with higher TG concentration (*β* = 0.011, *P* = 0.021) and lower HDL-C level (*β* = −0.039, *P* = 0.021). However, we did not observe any significant association between the level of DNA methylation in intragenic enhancer region of *CPT1A* and these phenotypes (*P* > 0.05).

**Table 5:** The associations of main phenotypes with the DNA methylation.

Indicators	*INSR*			*CPT1A*		
	*β*	*R* ^2^	P-value	*β*	*R* ^2^	*P*-value
BMI	0.000	<0.001	0.851	−0.003	0.006	0.132
WC	0.000	0.001	0.596	−0.001	0.009	0.189
FPG	0.003	0.002	0.466	0.000	<0.001	0.913
TG	**0.011**	**0.023**	**0.021**	−0.004	0.003	0.452
LDL-C	−0.007	0.005	0.271	−0.006	0.003	0.446
HDL-C	**−0.039**	**0.023**	**0.021**	0.031	0.015	0.079
DBP	0.000	<0.001	0.892	0.000	0.002	0.513
SBP	0.000	0.001	0.608	0.000	0.001	0.673

Abbreviations: BMI, Body mass index; WC, Waist circumstance; FPG, Fasting plasma glucose; TG, Triglyceride; LDL-C, Low density lipoprotein cholesterol; HDL-C, High density lipoprotein cholesterol; DBP, Diastolic blood pressure; SBP, Systolic blood pressure; *INSR*, Insulin receptor; *CPT1A*, carnitine palmitoyltransferase 1A; *IGF2*, insulin growth factor 2. *β*, regression coefficients from linear regression models. *R*^*2*^, coefficient of determination; Significant associations were depicted in bold print. Linear regression was used to analyze the associations between DNA methylation and main phenotypes.

## Discussion

To our knowledge, the current study is the first time to examine the association between prenatal exposure to the China famine and the level of DNA methylation in growth and metabolic related genes. We found that people exposed to the China famine in fetal stage had a higher DNA methylation level for *INSR*. After stratified by province, we only found that only individuals who exposed to severely famine in their fetus showed elevated level of the DNA methylation. In addition, the DNA methylation in the intragenic enhancer region of *INSR* was associated with the level of serum TG and HDL-C. These findings indicate that fetus exposure to the China famine exposure may be associated with altered DNA methylation level of *INSR* in later life, which underscores the importance of early-life interventions in reducing the disease burden in later life.

The development origins hypothesis speculated that adverse conditions in early life contributes to the elevated risk of adulthood diseases. Although the mechanism underlying these associations are still unclear, the involvement of epigenetic dysregulation has been proposed^25–27^. Previous animal model experiments have found protein restricted diet during pregnancy leads to a persistent change in average DNA methylation of certain genes in offspring, and result in the permanent change in certain phenotypes, such as coat color and tail type^15,28^. The current study observed that prenatal exposure to the China famine was associated with the changed DNA methylation level of *INSR*, which further provided direct human evidence in elaborating this hypothesis.

In the present study, we observed that the methylation level of *INSR* in individuals exposed to the China famine in their fetus was 7.6% higher than that in those who born in pre-famine or post-famine. It was consistent with findings of Dutch famine study, which observed people exposed to the famine in fetal stage had a 4.6% higher DNA methylation level than their non-exposed same-sex sibling^19^. In addition, the Dutch study also found that prenatal exposed to the famine was associated with the higher DNA methylation level in intragenic enhancer region of *CPT1A*^19^. However, that was not detected in the current study. We speculated that the differences in ethnicity and severity of the famine between the China famine and the Dutch famine could contribute to the inconsistent association. The China famine occurred in Asian populations. However, the Dutch famine occurred in European populations. Moreover, the China famine lasted for three years, affected almost 600 million population and led to about 30 million premature death^10^. However, the Dutch famine only lasted for 6 months^29^. Thus, subjects came from the Dutch famine exposed to famine less than six months during fetal stage. However, subjects came from the China famine exposed to famine throughout the entire fetal period. Additionally, Bangladesh famine began in July 1974, and end in June 1975 due to a severe monsoon in 1974 destroyed the majority of the annual rice crop in Matlab^21^. Thus, the severity and lasting time are less than the China famine.

Region-specific association was also performed and we found that individuals who exposed to the severely famine affected region (Anhui province) had a significant higher DNA methylation of *INSR*, but not in the less severely affected region (Jiangxi province). The excess mortality in famine period (1959–1961) compared to the pre-famine period (1956–1958) was used to reflect the severity of famine exposure. The excess mortality in Anhui province was 474.9%, but it was only 36.8% in Jiangxi province. Thus, a dose-response relationship between prenatal famine exposure and DNA methylation of *INSR* maybe exist, though further study is needed to conform this association.

In addition, we observed that DNA methylation level in intragenic enhancer region of *INSR* was positively associated with TG concentration, and negatively associated with HDL-C concentration. Moreover, severe famine exposure individuals seem to consume more meat than non-exposed individuals. These findings indicated that the severe famine plus later life eating habits might regulate epigenome. These associations were not observed in the study of Dutch famine, which did not observe association between DNA methylation in intragenic enhancer region of *INSR* and TG or HDL-C concentration, but it observed that DNA methylation at intragenic enhancer region of *CPT1A* was associated with LDL-C (*R*^2^ = 0.077, *P* < 0.01)^19^. However, we did not observe similar association between DNA methylation in *CPT1A* gene intragenic enhancer region and LDL-C. Although the DNA methylation was measured in peripheral whole blood, the DNA methylation patterns in peripheral whole blood could reflect the methylation patterns in other relevant tissues. Because the differential methylation induced by early development could be reserved through mitotic inheritance, which has been reported in model organisms^30,31^ and in human^32^. All the region measured in our study did not overlap with tissue differential methylation regions (DMRs) (identifying in a comprehensive WGBS data set), indicating that DNA methylation patterns in these regions could be reserved across other tissues, including liver or fatty tissues that involved in metabolism. This possibly explains the reason why prenatal famine exposure was associated with the DNA methylation in whole blood.

Although the current study only observed that waist circumference was significant higher in the famine exposure group than the non-exposed group, the adverse effect of the China famine on health condition might be great, which have been documented by our previous studies^11,13^. These studies found that early-life the China famine exposure significantly increased the level of TC, LDL and blood pressure. However, we did not observe consistent associations in the current study. We speculated that sample size might be inadequate to test these differences in this study.

Several limitations of the current study should be mentioned. Firstly, the DNA methylation was measured in peripheral whole blood, which may differ from the methylation patterns of other relevant tissues or organs. However, several model organisms studies^30,31^, as well as a recently in human study^32^, have found that differential methylation in whole blood may be similar with other tissues though mitotic inheritance reserves the methylation patterns induced in early development. Secondly, the DNA methylation was measured over five decades after exposure in the current study. We cannot assess whether the initially methylation differences between the prenatal famine-exposed group and non-exposed group waned or arisen over five decades. However, the Dutch famine studies have found that age did not affect the methylation patterns and observed that the changed DNA methylation patterns at *IGF*2 induced by early-life adverse conditions persist throughout life^19,20^. In addition, the associations of prenatal famine exposure with prenatal malnutrition-associated differentially methylated regions were independent of current lifestyle^19^. Thus, we speculated that the changed DNA methylation patterns induced by prenatal exposed to the China famine may reserve across five decades. Thirdly, the prenatal malnutrition-associated differentially methylated regions may be affected by environments and lifestyle in later time. However, we did not observe significant differences in lifestyle factors between exposed group and non-exposed group in the present study. Fourth, the residual confounding of dietary intake, drinking and smoking status on the association might still exist due to dichotomous variables were used as adjustment variables. Fifth, different genetic backgrounds between prenatal famine exposure individuals and non-exposed individuals might have an effect on the association between prenatal exposed group and non-exposed group. However, individuals were selected randomly from resident registration system, which could decrease the genetic backgrounds difference between two groups. Sixth, different nurturing environments between two group during young age could be also have effect on the association. The study is an observation study, and the data of eating habits during young age after birth cannot be obtained in the China famine. However, Health behavior during young age was associated with adulthood. Thus, we have controlled the adulthood frequency of dietary intake, physical activity level and smoking and alcohol use status, which could decrease the effects. Seventh, we couldn’t show the methylation change over time in the current study because we did not recruit the subjects who born between October 1st 1961 and September 30th 1962 because they might also exposure to famine during second or third trimester. Additionally, DNA methylation difference between exposed and unexposed groups might be derived from difference of cell composition in peripheral bloods. The DNA methylation levels in *INSR* is located in intron 9 of *INSR*, the region was differentially modified in chromatin state between blood cell types. This region is at “quiescent” and “poised promoter” state in T cells but at “enhancer” state in B cells, natural killer cells, and neutrophils^33^. However, the current study lacks the data of blood cell count. Though there have been these limitations, the current study explored the association of prenatal the China famine exposure with DNA methylation patterns over five decades for the first time and found that prenatal the China famine exposure was associated with elevated DNA methylation of *INSR*.

## Supplementary information

Supplementary materials

## Data Availability

The datasets generated during the current study are available from the corresponding author on reasonable request.
