# Supplementary material for: Early-Life Exposure to the Chinese Famine Is Associated with Higher Methylation Level in the INSR Gene in Later Adulthood
Source: Sci Rep. 2019 Mar 4;9:3354. doi: 10.1038/s41598-019-38596-6 (PMC6399294; doi:10.1038/s41598-019-38596-6)
Supplement: Supplementary file 1 — Supplementary materials [file 41598_2019_38596_MOESM1_ESM.docx]

**Early-Life Exposure to the Chinese Famine Is Associated with Higher Methylation Level in the *INSR* Gene in Later Adulthood**

Zhenghe WANG, Jieyun SONG, Yanhui LI; Bin DONG, Zhiyong ZOU, Jun MA

**Table S1 The detailed information of EpiTYPER primers**

| Target gene | Locus (hg18) | Primers | Strand | T(℃) | Sequences |
| --- | --- | --- | --- | --- | --- |
| *INSR* | chr19:7110130-7110574 | Forward (5'-3') | + | 58 | GGAGTTTTGTTTTGTTGTTAGGTTG |
|  |  | Reverse (5'-3') |  |  | CCTAAAAAATCCCTAAAATCCTCAA |
| *CPT1A* | chr11:68286513-68286952 | Forward (5'-3') | + | 60 | GTATTTTTGAAAGGGGTAGAGGAAA |
|  |  | Reverse (5'-3') |  |  | TACCAACCTAAACAACAACAAAACC |

Abbreviations: *INSR,* insulin receptor; *CPT1A*, carnitine palmitoyltransferase 1A.
